# Efflux Protein Expression in Human Stem Cell-Derived Retinal Pigment Epithelial Cells

**DOI:** 10.1371/journal.pone.0030089

**Published:** 2012-01-17

**Authors:** Kati Juuti-Uusitalo, Hanna Vaajasaari, Tuomas Ryhänen, Susanna Narkilahti, Riitta Suuronen, Eliisa Mannermaa, Kai Kaarniranta, Heli Skottman

**Affiliations:** 1 The Institute of Biomedical Technology, University of Tampere, Tampere, Finland; 2 Department of Ophthalmology, Institute of Clinical Medicine, University of Eastern Finland, Kuopio, Finland; 3 Department of Eye, Ear, and Oral Diseases, Tampere University Hospital, Tampere, Finland; 4 Department of Biomaterial Engineering, Tampere University of Technology, Tampere, Finland; 5 Department of Ophthalmology, Kuopio University Hospital, Kuopio, Finland; University of Torino, Italy

## Abstract

Retinal pigment epithelial (RPE) cells in the back of the eye nourish photoreceptor cells and form a selective barrier that influences drug transport from the blood to the photoreceptor cells. At the molecular level, ATP-dependent efflux transporters have a major role in drug delivery in human RPE. In this study, we assessed the relative expression of several ATP-dependent efflux transporter genes (*MRP1, -2, -3, -4, -5, -6, p-gp,* and *BCRP*), the protein expression and localization of MRP1, MRP4, and MRP5, and the functionality of MRP1 efflux pumps at different maturation stages of undifferentiated human embryonic stem cells (hESC) and RPE derived from the hESC (hESC-RPE). Our findings revealed that the gene expression of ATP-dependent efflux transporters *MRP1, -3, -4, -5,* and *p-gp* fluctuated during hESC-RPE maturation from undifferentiated hESC to fusiform, epithelioid, and finally to cobblestone hESC-RPE. Epithelioid hESC-RPE had the highest expression of MRP1, -3, -4, and P-gp, whereas the most mature cobblestone hESC-RPE had the highest expression of MRP5 and MRP6. These findings indicate that a similar efflux protein profile is shared between hESC-RPE and the human RPE cell line, ARPE-19, and suggest that hESC-RPE cells are suitable *in vitro* RPE models for drug transport studies. Embryonic stem cell model might provide a novel tool to study retinal cell differentiation, mechanisms of RPE -derived diseases, drug testing and targeted drug therapy.

## Introduction

Age-related macular degeneration (AMD) is a complex eye disorder and is the leading cause of blindness in developed countries. AMD has a multifactorial etiology and leads to a progressive loss of central vision in the elderly. The number of AMD patients is projected to double over the next few decades, becoming a major public health issue in the near future [1]. AMD is characterized by the degeneration of retinal photoreceptors rod and cones, retinal pigment epithelium (RPE), and Bruch's membrane, as well as detrimental alterations of the choroidal capillaries. One of the main functions of RPE cells is to nourish the neural cells, rod and cone cells. In senescent RPE cells, which are constantly exposed to oxidative stress, this ability is weakened, causing secondary adverse effects on the neural retina and ultimately leading to vision loss [2]. Thus, degeneration of the postmitotic RPE cells is one of the most important hallmarks of AMD.

The pathogenesis of AMD is complex and it has remained elusive. Therefore appropriate therapies have been difficult to establish. Only 20% of AMD patients, that have exudative form of disease, can be treated with intravitreal anti-VEGF injections. It is a huge challenge to develop new effective treatment alternatives for AMD. The most number of AMD patients are out of any treatments and exudative AMD cases load ophthalmological clinics by a new way that has created many problems to manage from injections in limited resources. One of the most interesting future treatment modality is certainly human pluripotent stem cell derived regenerative RPE cell therapy for AMD and other RPE -originated retinal diseases, such as retinitis pigmentosa [3], [4]. In addition these cells provide a potential resource as biological tool for drug discovery, toxicity screening and targeted drug therapy.

The polarized RPE cells constitute a polygonal monolayer between the neurosensory retina rod and cones and the fenestrated capillaries of the choroid. The RPE has multiple functions: absorption of light energy, transport of metabolites and nutrients between photoreceptors and choriocapillaris, expression of growth factors for photoreceptors, regulation of homeostasis of the ionic environment, phagocytosis of the shed tips of photoreceptor outer segments (POS), regulation of visual cycle, and creation of the blood–retinal barrier (BRB) [5]. The BRB is composed of two components: the outer part comprises the RPE and the inner part comprises the endothelial cells of the retinal vessels [6], [7]. Functionally, the RPE is very similar to the blood-brain barrier (BBB). Several membrane-associated transport proteins, such as P-glycoprotein (P-gp), multidrug resistance-associated proteins (MRPs), breast cancer resistance protein (BCRP), and organic anion transporting polypeptides, have been characterized at the BRB and BBB and play a major role in regulating tissue bioavailability of several pharmacologic agents [8], [9]. The nine MRPs (MRP1-MRP9) represent the majority of the 12 MRP subfamily members belonging to the 48 human ATP-binding cassette transporters [10], [11]. Cloning, functional characterization, and cellular localization studies have identified most MRP subfamily members as ATP-dependent efflux pumps with high substrate specificity for the transport of endogenous and xenobiotic anionic substances. Efflux pumps both regulate drug transport and affect tissue pathology [10], [11]. Our recent findings revealed that a similar efflux protein profile is shared between the human RPE cell line, ARPE-19, and bovine primary RPE cells. [6]

The ARPE-19 cell line, however, does not fully resemble the human RPE; therefore, more relevant human-derived RPE cells are needed as better in vitro models for drug testing and screening [12]. RPE-like cells have been successfully differentiated from human embryonic stem cells (hESC) and human induced pluripotent stem cells (hiPSC) [12], [13]. hESC and hiPSC-derived RPE cells (hiPSC-RPE) express genes and proteins corresponding to the human RPE [12], [14], [15]. In addition, strongly pigmented hESC-RPE cells are able to phagocytose photoreceptor outer segments, secrete RPE trophic factors, and form a tight epithelium with high resistance [15]–[17]. The hESC-RPE cells are suggested to be an excellent *in vitro* model of human RPE [12], but it is important to evaluate whether the properties of these in vitro-differentiated RPE cells are truly similar to those of human RPE. Here, we studied the expression and functionality of ATP-dependent efflux transporters in undifferentiated hESC and in hESC-derived pigmented RPE (hESC-RPE) cells at different maturation stages to evaluate whether hESC-RPE are useful for drug screening and toxicology studies.

## Materials and Methods

### Cell lines

We used the hESC line Regea08/017 previously derived in our laboratory [18] and the commercially available RPE cell line ARPE-19 as a control (American Type Culture Collection (ATCC), Manassas, VA).

**Figure 1:**
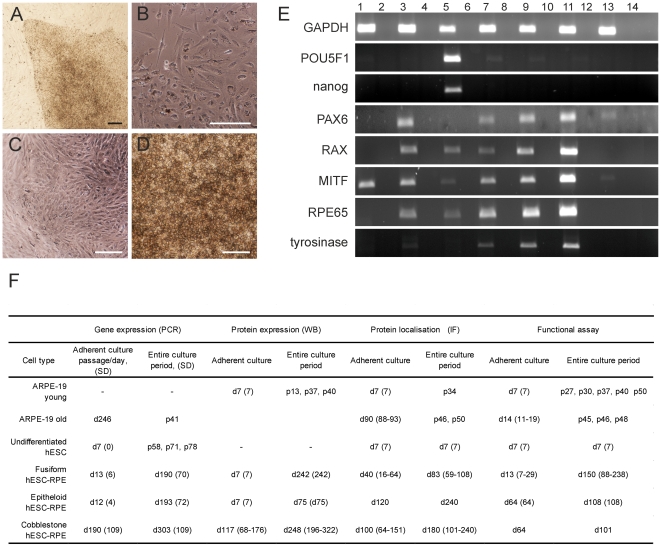
Morphology and gene expression of hESC on different maturation stages. Brightfield micrographs of cell cultures showing the representative morphology of **A**) undifferentiated hESC (Regea08/017), **B**) fusiform hESC-RPE, **C**) epithelioid hESC-RPE, **D**) cobblestone hESC-RPE. Scale bars, 100 µm, **E**) Gene expression of **1**: D407, **3**: ARPE-19, **5**: undifferentiated hESC, **7**: fusiform hESC-RPE, **9**: epithelioid hESC-RPE, **11**: cobblestone hESC-RPE, **13**: hFF. –RT- negative controls (i.e., samples not treated with reverse transcriptase) are placed adjacent to each sample in the same order: **2**: D407, **4**: ARPE-19, **6**: undifferentiated hESC, **8**: fusiform hESC-RPE, **10**: epithelioid hESC-RPE, **12**: cobblestone hESC-RPE, **14**: hFF. **F**) Culture periods of the studied samples in all analyses. Cells were selected based on their morphology rather than the culture period.

The hESC line Regea08/017 (46, XX), derived in our laboratory and characterized as previously described [18], was cultured on a mitotically inactivated (γ-irradiated, 40 Gy) human foreskin fibroblast (hFF) cell line (36 500 cells/cm^2^; CRL-2429, ATCC) at 37°C in 5% CO_2_ in hESC culture medium comprising Knock-Out Dulbecco's Modified Eagle Medium (KO-DMEM), 20% Knock-Out serum replacement (KO-SR), 2 mM GlutaMax, 0.1 mM 2-mercaptoethanol (all from Life Technologies, Carlsbad, CA, USA), 1% Minimum Essential Medium non-essential amino acids, 8 ng/ml human basic fibroblast growth factor (bFGF) (R&D Systems Inc., Minneapolis, MN, USA), and 50 U/ml Penicillin/Streptomycin (both from Cambrex Bio Science, Walkersville, MD, USA). The culture medium was replenished six times a week. The undifferentiated hESC (Fig. 1A) were passaged mechanically at 6 to 7-day intervals.

RPE cell differentiation was induced in floating cell aggregates by reducing the KO-SR concentration to 15% and removing the bFGF, as previously described [15]. The culture medium for the floating aggregates was changed three times a week.

Pigmented cells were manually dissected from the aggregates, and further dissociated with 1x Trypsin-EDTA before seeding on collagen IV- (5 µg/cm^2^; Sigma-Aldrich, St. Louis, MO, USA) coated wells of 24-well plates (NUNC, Thermo Fisher Scientific, Tokyo, Japan) or on BD Biocoat culture plate inserts (BD Biosciences, San Jose, CA). On adherent culture, pigmented cells underwent morphologic changes starting from a non-pigmented fusiform morphology (Fig. 1B) followed by rounding to more pigmented epithelioid cells (Fig. 1C), and finally developed a typical RPE-like cobblestone morphology (Fig. 1D). We selected the samples for RNA and protein extraction, immunofluorescence labeling, and functional testing based on their morphologic appearance (Fig. 1B–D), rather than the culturing time (Fig. 1F).

The commercially available ARPE-19 cell line was grown in Dulbecco's Modified Eagle Medium (DMEM-F12) (1∶1) supplemented with 10% fetal bovine serum (PAA Laboratories, Cölbe, Germany), 100 U/ml Streptomycin/Penicillin (both from Cambrex Bio Science). Cells were cultured in a 5% CO_2_ atmosphere at 37°C and subcultured on 25-cm^2^ cell culture flasks until they reached 80% confluency. For the experiments, ARPE-19 cells were enzymatically dissociated and seeded similarly as the hESC-RPE cells. The medium was changed three times a week. The culture periods are shown in Figure 1. A spontaneously transformed RPE cell line (D407), Human Embryonic Kidney 293 cells (HEK293), and hFF were used as control materials for the polymerase chain reaction (PCR) analyses. The RNA samples from D407 and HEK293, were the same reference RNA samples used previously [6].

### RNA isolation

Total RNA was isolated with NucleoSpin XS-kit (Macherey-Nagel, GmbH & Co, Düren, Germany) according to the manufacturer's instructions. The RNA concentration and the quality were assessed using a NanoDrop 1000 spectrophotometer (NanoDrop Technologies, Wilmington, DE, USA).

### Reverse transcription- (RT) PCR

RNA (40 ng) was reverse-transcribed using MultiScribe Reverse Transcriptase (Applied Biosystems, Foster City, CA, USA) according to the manufacturer's instructions in the presence of an RNase inhibitor. In addition, genomic control reactions excluding the restriction enzyme for each RNA sample were performed. Complementary DNA was used as a template in a following PCR reaction, which was carried out using 5 U/μl Taq DNA Polymerase (Fermentas, Thermo Fisher Scientific Inc., Leicestershire, UK) with 5 μM primers specific for particular genes (Biomers.net GmbH, Söflinger, Germany; Table 1). The PCR reactions were carried out in PCR MasterCycler ep gradient (Eppendorf AG, Hamburg, Germany) as follows: 95°C 3 min, 95°C 30 s, annealing 30 s, 72°C 1 min, 72°C 5 min, for 38 cycles. Annealing temperatures and primer sequences are presented in Table 1. PCR products were analyzed on 2% agarose gels with a 50-bp DNA ladder (MassRulerTM DNA Ladder Mix, Fermentas). The bands were visualized with the Quantity one 4.5.2. Basic program (Bio-Rad Laboratories, Inc., Hercules, CA, USA).

**Table 1:** Reverse-transcriptase–PCR primer sequences and used annealing temperatures.

Gene	Primer sequences (5′>3′)		T_ann_
	Forward	Reverse	
GAPDH	GTT CGA CAG TCA GCC GCA TC	GGA ATT TGC CAT GGG TGG A	55
POU5F1	CGTGAAGCTGGAGAAGGAGAAGCTG	AAGGGCCGCAGCTTACACATGTTC	62
nanog	TGCAAATGTCTTCTGCTGAGAT	GTTCAGGATGTTGGAGAGTTC	55
PAX6	AAC AGA CAC AGC CCT CAC AAA CA	CGG GAA CTT GAA CTG GAA CTG AC	60
RAX	CTG AAA GCC AAG GAG CAC ATC	CTC CTG GGA ATG GCC AAG TTT	55
MITF	AAG TCC TGA GCT TGC CAT GT	GGC AGA CCT TGG TTT CCA TA	52
RPE65	TCC CCA ATA CAA CTG CCA CT	CAC CACC ACA CTC AGA ACT A	52
tyrosinase	TGC CAA CGA TCC TAT CTT CC	GAC ACA GCA AGC TCA CAA GC	52

### Quantitative RT-PCR

Relative gene expression comparisons were performed using quantitative RT-PCR (qRT-PCR). FAM-labeled TaqMan Gene Expression Assays (Applied Biosystems) were used for the following genes: *MRP1* (Hs00219905_m1), *MRP2* (Hs00166123_m1), *MRP3* (Hs00358656_m1), *MRP4* (Hs00195260_m1), *MRP5* (Hs00981071_m1), *MRP6* (Hs00184566_m1), *P-gp* (Hs00184500_m1), and *BCRP* (Hs01053790_m1). RNA (200 ng) was reverse transcribed to cDNA as described above. The synthesized cDNA was diluted 1∶5 in RNase-free water and 3.0 µl was added to the final reaction (total 15 µl). No template controls were prepared for any of the genes. Reactions were carried out according to the manufacturer's instructions. The cDNAs were multiplied using Applied Biosystems 7300 Real-time Sequence Detection System: 2 min at 50°C, 10 min at 95°C, and 40 cycles repeating denaturation 15 s at 95°C, and annealing for 1 min at 60°C. qPCR analyses were performed from three individual biologic experiments, each reaction prepared as technical triplicates. Threshold cycle (Ct) values were determined using 7300 System SDS Software (Applied Biosystems) and data were further analyzed with Microsoft Office Excel 2003 (Microsoft Corporation, Redmond, WA). Relative gene expression was calculated using the 2-ΔΔCt method [19]. An internal control, glyceraldehyde 3-phosphate dehydrogenase (GAPDH, Hs99999905_m1), was used to normalize the data. The expression levels of either D407 or HEK293 were used as a reference depending on the gene studied. The technical replicate reactions were considered reliable if the standard deviation of the triplicate Ct values was less than 0.5.

### Western blotting

The cell samples washed with phosphate buffered saline (PBS, Lonza Group Ltd., Basal, Switzerland) and cells were lysed in M-PER lysis buffer (Thermo Scientific, Waltham, MA, USA) according to the manufacturer's instructions. Protein concentrations of samples were analyzed using the Bradford method [20]. The amount of ARPE-19 and hESC samples was 10 µg. The samples were run in 7% sodium dodecyl sulfate polyacrylamide (SDS-PAGE) gels and then wet-blotted to nitrocellulose membranes (GE Healthcare, Little Chalfont, Buckinghamshire, UK). Blocking was done with 3% fat-free dry milk in 0.3% Tween 20/PBS at room temperature (RT) for 1 h. Thereafter, membranes were incubated in primary antibody dilutions anti-MRP1 (1∶2000, overnight at 4°C), anti-MRP4 (1∶5000, 1 h at RT), or anti-MRP5 (1∶2000, 1 h at RT) rat monoclonal MRP antibodies (all from Abcam, Cambridge, UK) or alpha-tubulin (1∶4000, 30 min at RT, Sigma-Aldrich) that was used as a loading control. All primary antibodies were diluted in 0.5% bovine serum albumin (BSA) in 0.3% Tween 20/PBS. After 3×5 minutes washes with 0.3% Tween 20/PBS the membranes were incubated in horseradish peroxidase-conjugated anti-mouse IgG or antibody (GE Healthcare), diluted in 3% fat-free dry milk in 0.3% Tween 20/PBS (1∶10 000 for MRP1, 1∶10 000 for MRP4, and 1∶2000 for MRP5) for 1 h at RT, and 30 min at RT for alpha-tubulin (1∶10 000). Protein-antibody-complexes both in MRP and alpha-tubulin labeling were detected using an enhanced chemiluminescence method (Millipore, Billerica, MA, USA).

### Immunostaining

The cells were labeled as described previously [15]. Briefly, the cells were washed 3x5 minutes with PBS, fixed 10 min with 4% paraformaldehyde (pH 7.4; Sigma-Aldrich), and washed with PBS. Cells were permeabilized in 0.1% Triton X-100/PBS (Sigma-Aldrich), for 10 min and thereafter washed with PBS. Nonspecific binding sites were blocked with 3% BSA (Sigma-Aldrich) in PBS at RT for 1 h. Primary antibody incubations were done in 0.5% BSA-PBS, with rat monoclonal anti-MRP-1 (1∶100), anti-MRP-4 (1∶100), and anti-MRP-5 (1∶50), with rabbit anti-microphthalmia-associated transcription factor (MITF, 1∶350), mouse anti-cellular retinaldehyde-binding protein (CRALBP, 1∶1000), or mouse anti-Na^+^/K^+^ ATPase (1∶50; all antibodies were from Abcam) for 1h. Thereafter cells were washed 3x with PBS. The secondary antibody incubations were done in 0.5% BSA-PBS with donkey anti-mouse IgG and goat anti-rabbit IgG (both Alexa Fluor 488), goat anti-mouse IgG and goat anti-rabbit IgG (both Alexa Fluor 568; all from Molecular Probes, Life Technologies, Paisley, UK) in a 1∶1500 for 1 h, following repeated PBS washings. Nuclei were counterstained with 4′,6′-diamidino-2-phenylidole included in the mounting media (DAPI, Vector Laboratories Inc., Burlingame, CA). The entire labeling procedure was performed at RT. Confocal images were obtained with an LSM 700 confocal microscope (Carl Zeiss, Jena, Germany) using a 63× oil immersion objective and bright field images were obtained with an Olympus BX60 microscope (Olympus, Tokyo, Japan) with a 60× oil immersion objective with N.A. 1. Overlays and image processing of confocal images were done in ZEN-software (Carl Zeiss).

### Efflux activity test with calcein-AM

Calcein-AM is a substrate both for P-gp and MRP1 proteins [21], [22], thus MRP1 and P-gp efflux protein activity in the cells was assessed using the calcein–acetoxymethyl (AM) assay. Calcein-AM is cell permeable until it is metabolized by intracellular esterases to AM and calcein, a cell-impermeant fluorescent compound [6]. Efflux pump inhibitors either totally inhibit or slow the pumping rate, thus allowing the esterases more time to metabolize calcein-AM to calcein. The efficacy of inhibition is observed as an increase in intracellular fluorescence. The experiment was performed as previously described [6]. Briefly, the cells were pre-equilibrated with 25 mM HEPES-buffered Hank's balanced salt solution (pH7.4) with or without one of the following inhibitors, 15 µM cyclosporine A (Calbiochem, La Jolla, CA, USA), 200 µM progesterone (Sigma-Aldrich), 500 µM verapamil (ICN Biomedicals, Irvine, CA), or 100 µM MK571 (Cayman Chemicals, Ann Arbor, MI, USA) for 20 min at 37°C. Thereafter, calcein-AM (Calbiochem) was added to a final concentration of 2 µM and incubation continued for an additional 20 min at 37°C. Test solutions were changed to ice-cold buffer and intracellular fluorescence was measured using a Victor 1420 Multilabel Counter (Wallack, Finland) with excitation wavelength of 480 nm and an emission wavelength of 535 nm.

### Cell viability test

Cell viability was assessed from ARPE19 and hESC RPE cells simultaneously as the efflux pump activity test with the Live/Dead Viability/Cytotoxity kit for Mammalian cells (Invitrogen). Briefly, the cells were rinsed with DPBS and incubated at RT for 40 min with a mixture of 0.25 µM Calcein AM (green fluorescence) and 0,5 µM Ethidium homodimer-1 (red fluorescence, EthD-1). A fluorescence microscope (Olympus IX) was used to image the viable cells (green fluorescence) and dead cells (red fluorescence) with 10x long working distance objective.

### Statistical analyses

Statistical analysis of the qRT-PCR data was performed using analysis of variance (ANOVA) with Bonferroni's correction for multiple comparisons, and the calcein-AM assay with a one-sample t-test, both with PASW Statistics, version 18. P-values of less than 0.05 were considered statistically significant and P values of less than 0.01 were considered highly significant.

### Ethical issues

We have approval from the National Authority for Medicolegal Affairs Finland research with human embryos (Dnro 1426/32/300/05) and a supportive statement was obtained from the local ethics committee of the Pirkanmaa hospital district Finland to derive and expand hESC lines from surplus embryos not used in the treatment of infertility by the donating couples, and to use these lines for research purposes (R05116). No new cell lines were derived in this study.

## Results

### hESC-derived RPE cells express eye-specific genes

RT-PCR analysis was used to assess the cell maturation status. Pluripotency genes *POU5F1* and *nanog* typically expressed by undifferentiated hESC were expressed only by undifferentiated hESC, as expected (Fig. 1E). *PAX6,* one the first markers of the development of the neuroectoderm and eye, and eye-specific genes *RAX*, *MITF* and *RPE65* were expressed by both ARPE-19 and hESC-RPE cells at all maturation stages (i.e., fusiform, epithelioid, and cobblestone hESC-RPE). Undifferentiated hESC also expressed the eye-specific genes at very low levels. *Tyrosinase*, which is important for melanin synthesis, was expressed by hESC-RPE cells but not in ARPE-19 or D407 cells. None of the analyzed eye-specific genes, except for *MITF*, were detected in D407 cells. In addition, none of the studied genes, except for GAPDH, and faint expression of *PAX6,* were detected in hFF, which was analyzed as a possible source of background expression for the undifferentiated hESC samples.

### Efflux protein gene expression during RPE cell differentiation

The relative expression of several ATP-dependent efflux transporters (*MRP1, -2, -3, -4, -5, -6, p-gp,* and *BCRP*) was examined with qRT-PCR. The mRNA expression levels in the spontaneously transformed cell line D407 were used as a reference sample for all other studied genes except the *MRP6* gene, which has not been previously detected in D407 cells, and thus HEK293 cells were used as reference for *MRP6* gene expression studies.

**Figure 2:**
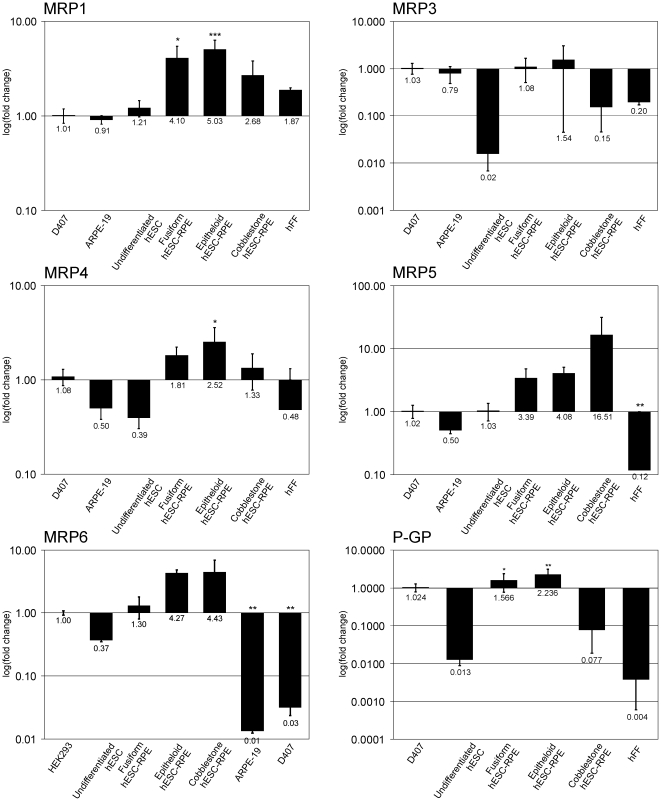
Expression of ATP-dependent efflux transporter genes. Relative expression of MRP1, MRP3, MRP4, MRP5, P-gp, and MRP6 genes. D407 used as reference sample for all genes except MRP-6, for which HEK-293 was used instead. Values that are significantly different from those of the reference sample are marked with an asterisk (*). For better visualization, fold-change is represented on a logarithmic scale. Standard deviations of fold-change from three separate experiments are presented as error bars.

During all maturation stages, hESC-RPE cells expressed higher levels of *MRP1* gene than D407 cells (Fig. 2). *MRP2* gene expression levels were significantly lower in all studied cells than in D407 (see Supplementary Table S1). Fusiform and epithelioid hESC-RPE cells expressed equal amounts of the *MRP3* as D407, while undifferentiated hESC, cobblestone hESC-RPE cells, and hFF expressed lower levels of the *MRP3* gene than D407 cells (Fig. 2). Fusiform, epithelioid, and cobblestone hESC-RPE expressed higher levels of the *MRP4* gene than D407 cells. *MRP5* was expressed in hFF cells and ARPE-19 lower levels, and undifferentiated hESC expressed similar amounts of *MRP5* gene as D407 cells. Conversely mature hESC-RPE cells expressed higher levels of the *MRP5* gene than D407 cells. The *MRP6* gene expression level was very low in D407 and ARPE-19 cells; therefore, HEK293 cells were used as a reference sample (Supplementary Table S1). Fusiform hESC-RPE expressed the *MRPP6* gene at the same level as HEK293. The MRP6 expression levels were much higher in epithelioid and cobblestone hESC-RPE cells than in HEK293 cells, and much lower in undifferentiated hESC, ARPE-19, and D407 cells than in HEK293 cells (Fig. 2). Fusiform and epithelioid hESC-RPE expressed significantly higher levels of the *p-gp* gene than D407 cells, whereas the *p-gp* gene was undetectable in ARPE-19 cells. In addition, the undifferentiated hESC, cobblestone hESC-RPE, and hFF expressed lower levels of the *p-gp* gene than D407 cells (Fig. 2) (Supplementary Table S1). *BCRP* gene expression levels in all studied samples were significantly lower than those in D407 cells.

### Efflux protein expression

**Figure 3:**
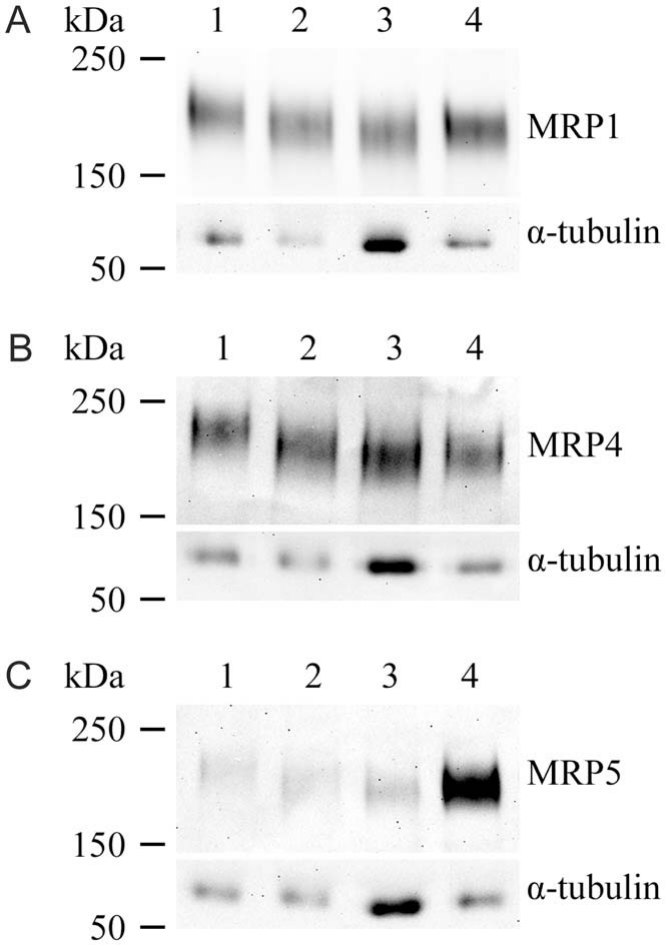
Expression of ATP-dependent efflux transporter proteins. Expression of MRP1 (A), MRP4 (B), and MRP5 (C) proteins in ARPE-19 cells (lane 1), fusiform hESC-RPE (lane 2), epithelioid (lane 3) and cobblestone (lane 4) hESC-RPE (lanes 4) detected with Western blotting. Alpha-tubulin was used as the loading control.

To ensure that the gene transcripts were translated to proteins, we used Western blot to examine whether the ARPE-19 and fusiform, epithelioid and cobblestone hESC-RPE expressed MRP1, MRP4, and MRP5 protein. Both ARPE-19 and the hESC-RPE cells produced MRP1 (Fig. 3A), -4 (Fig. 3B), and -5 (Fig. 3C) proteins. The MRP1 protein expression slightly increased and MRP5 protein expression was extensively increased during the maturation process (Fig. 3A), whereas MRP4 expression remained stable during the maturation.

### Efflux protein localization during RPE cell differentiation

**Figure 4:**
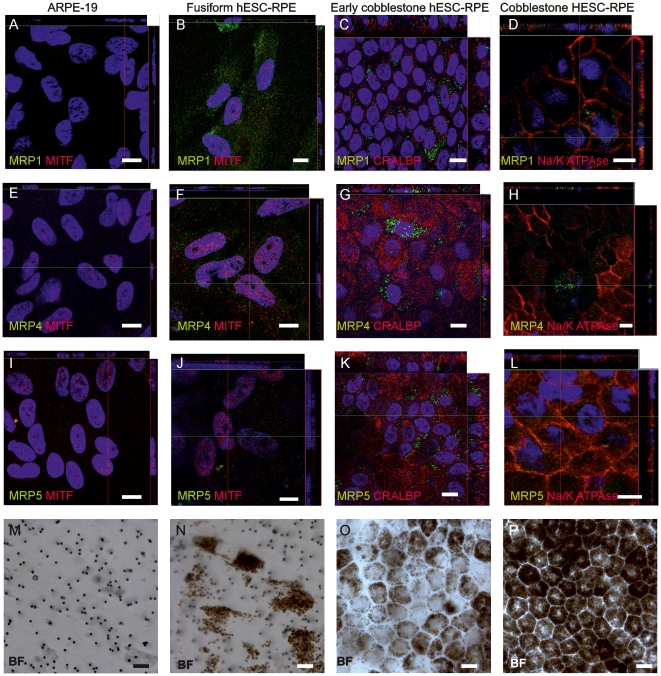
Localization of ATP-dependent efflux transporter proteins. A-L ) Confocal micrographs after indirect immunofluorescence labeling with efflux pump proteins MRP-1, -4, or -5 (green), and eye-specific proteins MITF and cellular retinaldehyde-binding protein (CRALBP, both red), the polarization marker Na^+^/K^+^ ATPase (red), and the nuclear label 4′,6′-diamidino-2-phenylidole (blue). In figures **M-P**) the brightfield micrographs show the same ARPE-19 cells and fusiform, early cobblestone, and cobblestone hESC-RPE as shown in the confocal images. Scale bars, 10 µm.

The cellular localization of MRP1, -3, and -5 proteins was assessed in ARPE-19 cells and in fusiform, epithelioid, and cobblestone hESC-RPE (Fig. 4A–P). The overall labeling intensity with MRP antibodies was extremely low. None of the studied MRPs were detected in ARPE-19 cells (Fig. 4A, E, I). The fusiform hESC-RPE had low but still detectable amounts of MRP1 and MRP4 and a very low amount of MRP5 protein staining (Fig. 4B, F, J). The early cobblestone hESC-RPE had detectable amounts of subcellularly localized MRP1, -4, and -5 proteins (Fig. 4C, G, K). The cobblestone hESC-RPE cells had MRP1, -4, and -5 protein staining that coincided with apical Na^+^/K^+^ ATPase staining (Fig. 4D, H, L).

### Functionality of MRP1 efflux pump during RPE cell differentiation

**Figure 5:**
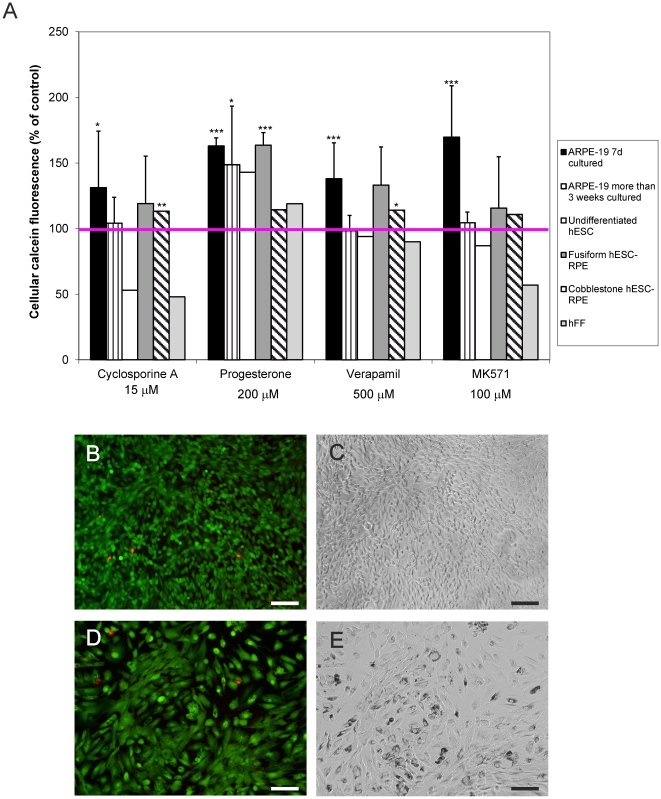
Functional testing of ATP-dependent efflux transporter proteins and viability of cultured cells. A ) Calcein retention in ARPE-19, undifferentiated hESC, fusiform, and cobblestone hESC-RPE, and hFF cells in the presence or absence ( =  control) of efflux protein inhibitors. Retention is expressed as a percentage of fluorescence relative to control (control = 100%). The studies were repeated at least three times for ARPE-19 and fusiform hESC-RPE, and once each for undifferentiated hESC, cobblestone hESC-RPE, and hFF. Data are expressed as mean±SD, *p<0.05, **p<0.01, ***p<0.001. B-E) Representative images of viable (green fluorescence) and dead (red fluorescence) ARPE19 (B,C) and fusiform hESC RPE cells (D,E). The scale bar 100 µM.

Efflux protein activity in the cells was assessed with Calcein-AM assay from ARPE-19 cells and in fusiform, epithelioid, and cobblestone hESC-RPE. ARPE-19 cells cultured for 7 days showed efflux activity, but the activity was lost when the cells were cultured for longer periods of time (Fig. 5A). On the other hand, fusiform hESC-RPE cells had higher MRP1 activity than cobblestone hESC-RPE. The undifferentiated hESC and hFF had no MRP1 efflux pump activity.

### Cell viability

Microscopic observations revealed that after 7 days of culture, at the time of functionality tests, both ARPE19 (Fig. 5B and C) and fusiform hESC RPE (Fig. 5D and E) cells were viable, and the number of dead cells was low.

## Discussion

Currently there is no curative treatment for exudative AMD, therefore human pluripotent stem cell derived RPE cells are highly desirable source of cells for cell therapy in AMD [3], [4]. Furthermore these cells offer a biological tool for drug discovery, toxicity screening and targeted drug therapy. For that purpose we have assessed the expression status and function of ATP-dependent efflux transporters in stem cell derived RPE cells.

Before examining the expression status of ATP-dependent efflux transporters, we assessed the maturation status of the samples using RT-PCR, which revealed that the spontaneously transformed retinal cell line, D407, previously used in efflux transporter studies [6], [23], expressed no eye-specific genes other than *MITF*. Eye-specific gene expression was detected in ARPE-19, which is a cell line that is widely used for RPE drug transport studies [9], [24], confirming that ARPE-19 cells are a good RPE standard.

Human ESC-RPE cells at all maturation stages (fusiform, epithelioid, and cobblestone) expressed eye-specific genes, as expected. Furthermore, the expression of *PAX6, RAX, RPE65*, and tyrosinase increased from fusiform to epithelioid and from epithelioid to cobblestone hESC-RPE, confirming that classification according to morphology is valid.

MRP1 protein is predominantly expressed in human cells that form blood-tissue barriers [10], [25]. Several xenobiotics, dietary, and synthetic flavonoids (e.g., fruit pigments) modulate the MRP1 pump [25]. MRP1 expression is detected in human RPE [26], primary RPE cells [27], and RPE cell lines [6], [27]. The present study showed for the first time that hESC-derived RPE cells also express MRP1 at both the mRNA and protein levels. *MRP1* mRNA expression clearly peaked in the early stages of differentiation in fusiform-shaped cells, and declined thereafter in fully mature cells with a cobblestone morphology. These results are consistent with those of previous studies [6], [26], [27], although this is the first study to confirm that *MRP1* expression fluctuates depending on the maturation status. The fluctuation in expression was also observed in an efflux pump functional test with calcein-AM. In the functional test, epithelioid cells had higher activity than cobblestone cells, and undifferentiated hESC had no activity at all. Graff and coworkers [28] as well as Rao and coworkers [29] previously reported that MRP1 localizes on the apical side of the BBB. In the present study, the localization of MRP1 changed when hESC-RPE cells matured: in fusiform cells, MRP1 was located primarily subcellularly with only faint expression in cellular projections in the majority of cells; in early cobblestone MRP1 was located intracellularly near the nucleus; and in mature cells with a cobblestone morphology, MRP1 was located on the apical side of the polarized cells. The change in the amount and localization of MRP1 might also reflect the differences in the MRP1 function in native RPE. The overall intensity in immunofluorescence labeling was very low, even in hESC-RPE cells that expressed high amounts of MRP1 protein, therefore the labeling in ARPE-19 cells might have remained below the detection level.

In previous studies, MRP2 expression was detected in RPE extracted from cadaveric eyes [26] and in D407, but not in ARPE-19 cells [6]. Our qRT-PCR expression analysis showed that hESC and ARPE-19 cells expressed only very low levels of *MPR2* gene compared to D407. Thus, our results are consistent with the previous results obtained with ARPE-19 cells [6].

MRP3 gene expression has been previously detected in human retina/choroid [30] and in D407 cells, but not in ARPE-19 cells [6]. MRP3 expression fluctuated during the hESC-RPE cell maturation: MRP3 expression was very low in undifferentiated hESC, but the expression was increased from fusiform hESC-RPE to epithelioid hESC-RPE and diminished again in cobblestone hESC-RPE.

MRP4 is an ATP-dependent organic anion transporter [10] that has a role, for example, in prostaglandin transport [31] in the eye. It also interacts with many drugs, such as 5′fluorouracil, zidovudine, ganciclovir, and vincristine that are used to treat retinal conditions [32]–[34]. In earlier studies MRP4 expression was detected in human retinal samples [26] and in ARPE-19 and D407 cells [6]. In the present study, MRP4 expression was low in undifferentiated hESC, but was induced when cells matured to fusiform hESC-RPE. MRP4 protein localizes either on the basolateral or apical membrane of the cells, depending on the cell type [35], nevertheless, the localization of MRP4 has not been previously studied in RPE cells. The microscopic examination revealed that the fusiform cells were weakly MRP4 positive, and the positivity was scattered within the cell. In highly pigmented cobblestone hESC-RPE cells, MRP4 protein was localized on the apical side near the Na^+^/K^+^ ATPase-expressing cells. The overall labeling intensity in immunofluorescence labeling was very low, and MRP4 labeling in ARPE-19 cells probably remained below the detection level.

MRP5 has a broad substrate and inhibitor specificity [10]. In eye diseases, MRP5 has an important role as it interacts with drugs, such as Etoposide, used for treatment of retinoblastoma [36]. MRP5 expression has also been linked to AMD development, and its expression decreases in RPE cells cultured on old Bruch's membrane [37]. Both the MRP5 gene and protein are expressed in D407 and ARPE-19 cells [6], [37]. Here, MRP5 expression increased both in mRNA and protein level during hESC-RPE cell maturation, which is consistent with a previous study [37]. MRP5 is apically localized in the BBB [38], but in the BRB the site of expression had not been previously determined. In immunofluorescence labeling, MRP5 was not detected in ARPE-19 cells and very few subcellular signals were observed in fusiform cells. In highly pigmented cobblestone hESC-RPE, the MRP5 protein localized to the apical side of the cells.

MRP6 gene expression has not been previously detected neither in native RPE [39] nor in ARPE-19 and D407 cell lines [6], although MRP6 gene ablation in mice increases the calcification of retina and Bruch's membrane [40]. Interestingly, the hESC-RPE cells expressed MRP6 at similar level to HEK293, and the expression increased during maturation of the hESC-RPE cells, whereas very low expression was detected in ARPE-19 and D407 cells. The discrepancy in the MRP6 expression between hESC-RPE and cadaveric RPE might be due to the fact that hESC-RPE are in a different maturation state than native RPE or that MRP6 is expressed in very low levels in native RPE and thus remains undetected.

P-gp is expressed in human [41] and porcine [42] RPE, and at low levels in the h1RPE cell line derived from immortalized primary RPE cells [23] and in ARPE-19 cells [6], [23]. In the present study, P-gp gene expression levels peaked in immature hESC-RPE. Thus, the expression pattern of p-gp was similar to that of MRP1 and MRP4. BCRP is expressed in human RPE [26] and in D407, but not in ARPE-19 cells [6]. BCRP expression was very low in ARPE-19 and hESC-RPE.

In conclusion, the findings of the present study clearly demonstrated that expression of genes for the ATP-dependent efflux transporters MRP1, -3, -4, -5, and P-gp fluctuates in undifferentiated hESC and hESC-RPE at different maturation stages. In addition, based on the gene expression profile, hESC-RPE cells more closely resemble ARPE-19 cells than D407 cells, suggesting that hESC-RPE cells have important RPE cell-like properties, which make these cells an excellent in vitro cell model for drug transportation studies for AMD drug testing and development.

## Supporting Information

Table S1 Gene expression data with standard deviations (SD) and calculations of statistical significance (p).
(DOC)
