# Supplementary material for: Efflux Protein Expression in Human Stem Cell-Derived Retinal Pigment Epithelial Cells
Source: PLoS One. 2012 Jan 17;7(1):e30089. doi: 10.1371/journal.pone.0030089 (PMC3260202; doi:10.1371/journal.pone.0030089)
Supplement: Table S1 — Gene expression data with standard deviations (SD) and calculations of statistical significance (p). (DOC) [file pone.0030089.s001.doc]

**Supplemental Table 1.**

Gene expression data with standard deviations (SD) and calculations of statistical significance (p).

|  |  |  |  |  |  |  |
| --- | --- | --- | --- | --- | --- | --- |
| Gene of interest | Cell line | Average Ct (GAPDH) | Average Ct (gene of interest | Average fold change (2-ddCt) | SD (fold change) | p value (fold regulation) |
| **MRP1** | **D407** | **18,63** | **25,16** | **1,01** | **0,174** |  |
| MRP1 | ARPE-19 | 18,43 | 25,11 | 0,91 | 0,098 | 1,000 |
| MRP1 | Undifferentiated hESC | 18,09 | 24,76 | 1,21 | 0,237 | 1,000 |
| MRP1 | Fusiform hESC-RPE | 19,38 | 24,32 | 4,10 | 1,362 | 0,036 |
| MRP1 | Epitheloid hESC-RPE | 19,56 | 24,19 | 5,03 | 1,284 | 0,000 |
| MRP1 | Cobbelstone hESC-RPE | 18,57 | 24,23 | 2,68 | 1,131 | 1,000 |
| MRP1 | hFF | 21,30 | 25,78 | 1,87 | 0,110 | - |
| **MRP2** | **D407** | **18,63** | **24,22** | **1,02** | **0,226** |  |
| MRP2 | ARPE-19 | 18,43 | 32,83 | 0,00 | 0,001 | 0,000 |
| MRP2 | Undifferentiated hESC-RPE | 18,09 | 33,49 | 0,00 | 0,001 | 0,000 |
| MRP2 | Fusiform hESC-RPE | 19,38 | 32,37 | 0,01 | 0,003 | 0,000 |
| MRP2 | Epitheloid hESC-RPE | 19,56 | 33,14 | 0,01 | 0,005 | 0,000 |
| MRP2 | Cobbelstone hESC-RPE | 18,57 | 33,53 | 0,00 | 0,002 | 0,000 |
| MRP2 | hFF | 21,30 | 33,85 | 0,00 | 0,000 | - |
| **MRP3** | **D407** | **18,63** | **25,77** | **1,03** | **0,268** |  |
| MRP3 | ARPE-19 | 18,43 | 25,99 | 0,79 | 0,311 | 1,000 |
| MRP3 | Undifferentiated hESC-RPE | 18,09 | 31,84 | 0,02 | 0,009 | 0,349 |
| MRP3 | Fusiform hESC-RPE | 19,38 | 27,04 | 1,08 | 0,579 | 1,000 |
| MRP3 | Epitheloid hESC-RPE | 19,56 | 27,52 | 1,54 | 1,496 | 1,000 |
| MRP3 | Cobbelstone hESC-RPE | 18,57 | 29,57 | 0,15 | 0,108 | 1,000 |
| MRP3 | hFF | 21,30 | 29,52 | 0,20 | 0,026 | - |
| **MRP4** | **D407** | **18,63** | **25,40** | **1,08** | **0,211** |  |
| MRP4 | ARPE-19 | 18,43 | 26,37 | 0,50 | 0,119 | 1,000 |
| MRP4 | Undifferentiated hESC-RPE | 18,09 | 26,88 | 0,39 | 0,092 | 1,000 |
| MRP4 | Fusiform hESC-RPE | 19,38 | 25,94 | 1,81 | 0,415 | 1,000 |
| MRP4 | Epitheloid hESC-RPE | 19,56 | 25,76 | 2,52 | 1,061 | 0,015 |
| MRP4 | Cobbelstone hESC-RPE | 18,57 | 25,72 | 1,33 | 0,555 | 1,000 |
| MRP4 | hFF | 21,30 | 26,51 | 0,48 | 0,831 | - |
| **MRP5** | **D407** | **18,63** | **26,15** | **1,02** | **0,236** |  |
| MRP5 | ARPE-19 | 18,43 | 26,95 | 0,50 | 0,061 | 1,000 |
| MRP5 | Undifferentiated hESC-RPE | 18,09 | 26,03 | 1,03 | 0,316 | 1,000 |
| MRP5 | Fusiform hESC-RPE | 19,38 | 25,62 | 3,39 | 1,413 | 1,000 |
| MRP5 | Epitheloid hESC-RPE | 19,56 | 25,49 | 4,08 | 1,012 | 1,000 |
| MRP5 | Cobbelstone hESC-RPE | 18,57 | 22,98 | 16,51 | 14,903 | 1,000 |
| MRP5 | hFF | 21,30 | 28,90 | 0,12 | 0,202 | - |
| **p-gp** | **D407** | **18,63** | **26,72** | **1,02** | **0,244** |  |
| p-gp | ARPE-19 | 18,43 | undetected | - | - | - |
| p-gp | Undifferentiated hESC-RPE | 18,09 | 32,95 | 0,01 | 0,004 | 1,000 |
| p-gp | Fusiform hESC-RPE | 19,38 | 27,38 | 1,57 | 0,800 | 1,000 |
| p-gp | Epitheloid hESC-RPE | 19,56 | 27,01 | 2,24 | 0,898 | 0,630 |
| p-gp | Cobbelstone hESC-RPE | 18,57 | 31,07 | 0,08 | 0,058 | 1,000 |
| p-gp | hFF | 21,30 | 36,56 | 0,00 | 0,003 | - |
| **BCRP** | **D407** | **18,63** | **26,65** | **1,01** | **0,201** |  |
| BCRP | ARPE-19 | 18,43 | 33,91 | 0,01 | 0,003 | 0,000 |
| BCRP | Undifferentiated hESC-RPE | 18,09 | 28,00 | 0,37 | 0,172 | 0,000 |
| BCRP | Fusiform hESC-RPE | 19,38 | 33,98 | 0,01 | 0,004 | 0,000 |
| BCRP | Epitheloid hESC-RPE | 19,56 | 34,82 | 0,10 | 0,271 | 0,000 |
| BCRP | Cobbelstone hESC-RPE | 18,57 | 34,17 | 0,05 | 0,095 | 0,000 |
| BCRP | hFF | 18,74 | 32,24 | 0,01 | 0,001 | - |
| **MRP6** | **HEK293** | **18,03** | **29,79** | **1,00** | **0,079** |  |
| MRP6 | Undifferentiated hESC-RPE | 17,36 | 30,56 | 0,37 | 0,022 | 1,000 |
| MRP6 | Fusiform hESC-RPE | 18,43 | 29,90 | 1,30 | 0,502 | 1,000 |
| MRP6 | Epitheloid hESC-RPE | 19,20 | 28,88 | 4,27 | 0,570 | 0,009 |
| MRP6 | Cobbelstone hESC-RPE | 18,21 | 28,05 | 4,43 | 2,504 | 0,006 |
| MRP6 | ARPE19 | 17,31 | 35,31 | 0,01 | 0,001 | - |
| MRP6 | D407 | 17,14 | 33,92 | 0,03 | 0,008 | - |
| MRP6 | hFF | 18,69 | 33,27 | 0,13 | 0,021 | - |
|  |  |  |  |  |  |  |
